# Safety and efficacy of endobronchial ultrasound-guided transbronchial biopsy of intrathoracic lymphadenopathy

**DOI:** 10.3389/fmed.2025.1736768

**Published:** 2025-12-05

**Authors:** Huizhen Yang, Dechang Kong, Xiaoju Zhang, Xiangnan Li, Quncheng Zhang, Ruijie Zhou

**Affiliations:** 1 Department of Respiratory and Critical Care Medicine, Henan Provincial People’s Hospital, People’s Hospital of Zhengzhou University, Zhengzhou, China; 2 Department of Respiratory and Critical Care Medicine, People’s Hospital of Henan University, People’s Hospital of Henan Province, Zhengzhou, China

**Keywords:** lymphadenopathy, EBUS-TBNA, EBUS-TBCB, EBUS-TBFB, biopsy, laser-assisted biopsy

## Abstract

**Introduction:**

In clinical practice, endobronchial ultrasound-guided transbronchial needle aspiration (EBUS-TBNA) often yields small-volume specimens that may be inadequate for full pathological assessment. To overcome this limitation, EBUS-guided transbronchial cryobiopsy (EBUS-TBCB) and transbronchial forceps biopsy (EBUS-TBFB) have been increasingly adopted. This study systematically evaluates their diagnostic performance and safety in intrathoracic lymph node lesions to inform clinical decision-making.

**Methods:**

This retrospective study included patients who presented to Henan Provincial People’s Hospital between July 2018 and August 2025 with mediastinal or hilar lymph nodes of a short-axis diameter ≥ 1 cm on chest computed tomography (CT) or with abnormally increased metabolic activity of lymph nodes on positron emission tomography (PET)-CT. All patients underwent EBUS-TBNA followed by either EBUS-TBCB or EBUS-TBFB. The study aimed to evaluate the diagnostic performance and safety of the combined use of EBUS-TBNA with EBUS-TBCB or EBUS-TBFB for assessing intrathoracic lymph node lesions.

**Results:**

A total of 211 patients were included in this study, of whom 204 received a definitive diagnosis through EBUS-TBNA combined with EBUS-TBCB or EBUS-TBFB. The sensitivity, specificity, positive predictive value, negative predictive value, and overall diagnostic accuracy in differentiating benign from malignant thoracic lymph node lesions were 96.92% (126/130), 100% (81/81), 100% (126/126), 95.29% (81/85), and 98.10% (207/211), respectively. Further analysis revealed no significant difference in diagnostic yield between the combination of EBUS-TBNA with EBUS-TBCB and that with EBUS-TBFB. However, for pulmonary sarcoidosis, the diagnostic rate of EBUS-TBNA combined with EBUS-TBCB was 96.67% (29/30), higher than that of EBUS-TBNA combined with EBUS-TBFB at 87.50% (14/16). Post-procedural hemoptysis was the most common complication, occurring in 4.74% (10/211) of patients. No severe complications such as major bleeding, mediastinal infection, respiratory distress, or hypoxemia were observed.

**Conclusion:**

EBUS-TBNA combined with EBUS-TBCB or EBUS-TBFB is a safe, minimally invasive technique that achieves a high diagnostic yield for intrathoracic lymph node lesions. By obtaining sufficient and structurally intact tissue specimens, this approach facilitates molecular pathology testing, supports the diagnosis of benign conditions, and enables accurate pathological classification of malignant diseases.

## 1 Introduction

EBUS-TBNA is a widely used technique for the diagnosis of intrathoracic lymph node lesions, utilizing real-time ultrasound guidance to perform transbronchial puncture and sampling (1, 2). Although EBUS-TBNA provides good diagnostic yield and procedural safety, it is often limited by insufficient specimen size, which restricts the acquisition of complete histological structures. Consequently, accurate pathological classification is sometimes compromised due to small sample volume and susceptibility to tissue compression or damage. These limitations are particularly evident in the precise subtyping of lung cancer, the diagnosis of rare malignancies, and the differentiation of benign conditions such as sarcoidosis and tuberculosis (2). With the rapid development of immunotherapy and targeted therapy, the treatment paradigm for lung cancer has undergone profound changes. This progress has underscored the urgent clinical need to obtain sufficient, high-quality tissue specimens that can support molecular pathology testing and provide reliable histological evidence for both benign and malignant diseases.

To improve diagnostic accuracy, various enhanced sampling techniques have been developed to obtain larger and more intact tissue specimens, thereby improving tissue adequacy and diagnostic utility. Among these methods, EBUS-TBCB and EBUS-TBFB have been increasingly adopted. Under endobronchial ultrasound guidance, and with electrocautery or laser-assisted bronchial window creation when necessary, biopsy forceps or cryoprobes are advanced directly into target lymph nodes for tissue acquisition. Compared with EBUS-TBNA, EBUS-TBCB and EBUS-TBFB provide larger, intact specimens, substantially improving the diagnostic yield for benign conditions such as sarcoidosis and for malignant conditions such as lymphoma. Previous studies have reported that electrocautery-assisted EBUS-TBFB yields higher-quality specimens and greater diagnostic efficiency than EBUS-TBNA (3, 4). Meta-analyses further support these findings: the overall diagnostic yield of EBUS-TBNA combined with EBUS-TBCB or EBUS-TBFB for thoracic lymph node lesions is 88.7%, compared with 69.5% for EBUS-TBNA alone (5). Notably, in sarcoidosis, the diagnostic yield of EBUS-TBCB or EBUS-TBFB reaches 93.8%, higher than the 59.6% achieved with EBUS-TBNA (5–7). In terms of safety, the complication rate of combined EBUS-TBNA with EBUS-TBCB or EBUS-TBFB is low (2.4%), with no serious adverse events reported (5).

Based on this evidence, the present study conducted a retrospective analysis of real-world data from our center to further evaluate the diagnostic performance and safety of EBUS-TBNA combined with EBUS-TBCB or EBUS-TBFB.

## 2 Methods

### 2.1 Study design

We conducted a retrospective analysis of 211 patients who underwent bronchoscopy at Henan Provincial People’s Hospital between July 2018 and August 2025. Eligible patients met the following criteria: (1) chest computed tomography (CT) showing mediastinal or hilar lymph node enlargement with a short-axis diameter ≥ 1 cm within the puncture range of EBUS-TBNA, or positron emission tomography (PET)-CT demonstrating abnormally increased lymph node metabolism requiring further diagnostic clarification; (2) no contraindications to bronchoscopy. Exclusion criteria were as follows: (1) severe cardiovascular disease, severe psychiatric disorder, active massive hemoptysis, or anesthesia intolerance contraindicating bronchoscopy; (2) coagulation dysfunction (platelet count < 20 × 10⁹/L), considered a relative contraindication for bronchoscopy; (3) intraoperative ultrasound revealing lesions adjacent to abundant vasculature or major vessels, where biopsy was deemed high-risk for bleeding.

### 2.2 Operating procedures

All patients fasted for at least 6 h prior to the procedure and received intravenous anesthesia. The anesthetic regimen included sufentanil (0.2 μg/kg), propofol (1.5–2.0 mg/kg), and rocuronium bromide (0.3 mg/kg) to ensure adequate analgesia, sedation, and muscle relaxation. Continuous electrocardiographic monitoring was performed throughout the procedure, and an artificial airway was established using a laryngeal mask, followed by mechanical ventilation through a T-joint extension tube. Initially, a conventional white-light bronchoscopy was performed to conduct a comprehensive examination of the airways. When endobronchial lesions were identified, bronchoalveolar lavage (BAL) and/or transbronchial lung biopsy (TBLB) were performed as clinically indicated. BAL was preferred when infectious or granulomatous disease was suspected, as it enabled microbiological evaluation and facilitated exclusion of infectious etiologies. TBLB was generally performed when mediastinal lymphadenopathy coexisted with parenchymal lung lesions, enabling concurrent sampling of lung parenchyma for a more comprehensive clinical assessment. The decision to perform BAL and/or TBLB was made by the bronchoscopist based on real-time bronchoscopic findings and clinical indication. Subsequently, an ultrasound bronchoscope (BF-UC260F-OL8 or BF-UC260FW; Olympus, Tokyo, Japan) was introduced. Under real-time ultrasound guidance, the lobar to segmental bronchi were examined sequentially, and mediastinal and hilar lymph nodes were evaluated according to the American Thoracic Society (ATS) lymph node map, in conjunction with preoperative imaging findings. The EBUS Doppler function was employed to detect perinodal blood flow, and lymph nodes with favorable safety profiles and accessibility were preferentially selected as puncture targets.

**Figure 1:**
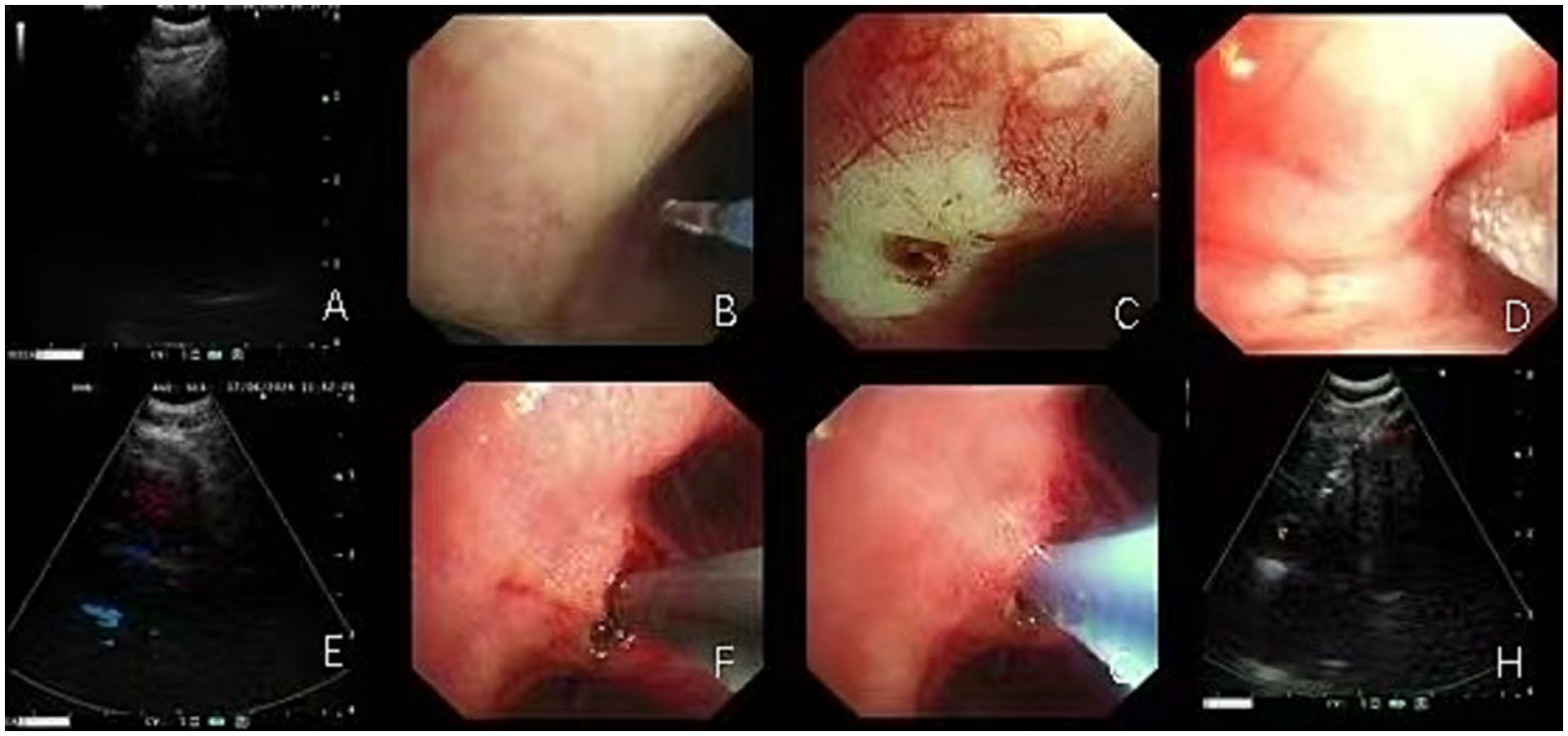
The lymph node biopsy procedure using EBUS-TBNA combined with EBUS-TBCB or EBUS-TBFB entails the use of biopsy forceps or cryoprobes, facilitated by electrocautery- or laser-assisted fenestration. **(A)** EBUS-TBNA biopsy of lymph nodes in the 4R group; **(B–E)** Sequential procedures showing EBUS-guided laser-assisted windowing followed by cryoprobe biopsy placement in 4R group lymph nodes; **(F–H)** EBUS-guided electrocautery-assisted windowing followed by biopsy forceps placement for biopsy in 11Rs lymph nodes.

EBUS-TBNA was performed under general anesthesia using a 21G puncture needle (Olympus, Tokyo, Japan). Under real-time ultrasound guidance, the target lymph nodes were punctured, and after confirming that the needle tip had entered the lesion, the needle was moved back and forth while applying negative pressure to obtain tissue cores (Figure 1A). Each lymph node was punctured at least four times and no more than seven times. Rapid on-site cytological evaluation (ROSE) was performed, with smear results independently assessed by a pathologist. In parallel, tissue cores were fixed in formalin and submitted for histological examination.

During EBUS-TBFB or EBUS-TBCB procedures, 1.5-mm biopsy forceps or 1.1-mm cryoprobes were introduced into the target lymph node via the tract established by EBUS-TBNA, with tissue samples obtained under real-time ultrasound guidance. In our center, the choice between TBCB and TBFB during EBUS-guided procedures followed predefined procedural criteria. Rapid on-site evaluation (ROSE) was routinely performed during EBUS-TBNA. When ROSE indicated malignancy or granulomatous inflammation but the histological specimen was insufficient, particularly when additional tissue was required to enable molecular testing, an additional biopsy using either TBCB or TBFB was undertaken. TBCB was performed when the target lymph node or lesion was adjacent to the airway wall, appeared firm on EBUS, or required a larger, intact tissue core for histopathological or molecular analysis (8). TBFB was selected for lesions near major vessels, for softer lesions on ultrasound, or when cryoprobe deployment was deemed unsafe due to anatomical constraints (9). The final decision was made by the bronchoscopist based on real-time EBUS findings and procedural safety considerations. For each lymph node station, 1–2 tissues were obtained for TBCB, and 2–3 forceps biopsy samples were taken for TBFB, depending on lesion characteristics and procedural factors (10, 11). The number of biopsies was based on tissue adequacy, lesion accessibility, and real-time safety assessment. Previous studies have reported that in approximately 28% of cases, biopsy instruments could not be advanced into the lymph node, most commonly due to limited penetration of the airway wall or nodal capsule (4, 12). In such cases, high-frequency electrocautery or laser-assisted window opening was employed to enlarge the entry site, thereby facilitating smooth passage of instruments into the target node (Figures 1B–H). This approach enabled the acquisition of larger and more structurally intact specimens, suitable for molecular pathology and immunohistochemical analysis (13).

After biopsy completion, a conventional bronchoscope was reintroduced to inspect the puncture sites and surrounding tissues for active bleeding. If bleeding was observed, local hemostatic measures were applied, including topical thrombin, iced saline lavage, or diluted norepinephrine.

### 2.3 Criteria for diagnosis

Benign and malignant tumors (including primary and metastatic) were diagnosed based on histopathological findings, which is considered the gold standard. Tissue specimens were obtained by bronchoscopic biopsy (e.g., EBUS-TBNA, EBUS-TBCB, or EBUS-TBFB, TBLB), surgical procedures, or other invasive techniques, and all cases were confirmed by the pathology department. The diagnostic criteria for lymph node tuberculosis included: (1) histopathological evidence, such as granulomatous inflammation with caseous necrosis or the presence of acid-fast bacilli; or (2) positive results from pathogen detection, including TB-DNA PCR, Xpert MTB/RIF, or metagenomic next-generation sequencing (mNGS). The diagnostic criteria for pulmonary sarcoidosis were: (1) histopathological evidence of non-caseating epithelioid granulomatous inflammation; (2) compatible clinical presentation and radiological findings; (3) exclusion of other granulomatous diseases such as tuberculosis or fungal infections.

Specimens obtained by the combined EBUS-TBNA and EBUS-TBCB or EBUS-TBFB approach were considered positive when malignant cells were detected and negative when no malignant cells were present. Diagnostic outcomes were further validated through subsequent clinical follow-up. Definitions were as follows: True positive (TP): cases in which malignancy was detected by the combined approach and subsequently confirmed by surgical or pathological evaluation, or by clinical follow-up. True negative (TN): cases in which a benign result from the combined approach was confirmed by surgical or pathological evaluation, or by clinical follow-up. False negative (FN): cases in which a benign result from the combined approach was later confirmed to be malignant by additional examination or clinical follow-up.

### 2.4 Postoperative adverse events

During and after EBUS-TBNA combined with EBUS-TBCB or EBUS-TBFB procedures, various adverse events or complications may arise from the procedure or patient-related factors. The primary complications are bleeding, infection, and pneumothorax. Complications related to EBUS-TBCB or EBUS-TBFB are similar to those of EBUS-TBNA, and these procedures are generally considered safe. The incidence and mortality rates are significantly lower than those of mediastinoscopy (14). The common complications and incidence rates reported in existing literature are as follows: cough approximately 14%, bleeding 10–14%, hemoptysis 2%, pneumothorax 1%, mediastinal emphysema 1%, and dyspnea 1% (15, 16). No cases of mediastinal infection were observed. Both mediastinal emphysema and pneumothorax typically resolved spontaneously without intervention. Salcedo Lobera et al. (17) reported that in their study of 50 patients, 2 cases developed hypoxemia and 1 case developed vocal cord hematoma. Symptoms improved after symptomatic treatment.

The grading of bleeding is based on the standards established by Ernst et al. and Yarmus et al. (18, 19), as follows: Level 0: No suctioning required; Level 1: Requires suctioning and bronchoscopic wedging for ≤ 2 min; Level 2: Requires bronchoscopic wedging for ≥ 3 min; Level 3: Requires local instillation of epinephrine or iced saline; Level 4: Requires hemodynamic support, including transfusion of blood products, selective main bronchial intubation, placement of bronchial occluders, hospital admission, or surgical intervention.

In clinical practice, most bleeding cases are mild (grades 0–1) and do not require special treatment. Bleeding usually resolves spontaneously with bronchoscopic suctioning or can be controlled by instillation of cold normal saline. If necessary, diluted norepinephrine solution can be applied topically for hemostasis.

### 2.5 Statistical analysis

Statistical analyses were conducted using IBM SPSS Statistics version 26.0 (IBM, Armonk, NY, United States). With the final pathological diagnosis or clinical follow-up as the reference standard, the sensitivity, specificity, positive predictive value, negative predictive value, and overall diagnostic accuracy of the combined EBUS-TBNA and EBUS-TBCB or EBUS-TBFB approach were calculated. Continuous variables with normal distribution are presented as mean ± standard deviation (mean ± SD) and compared using independent-sample t-tests. Non-normally distributed continuous variables are expressed as median and interquartile range (median [Q1, Q3]) and compared using the Wilcoxon rank-sum test. Categorical variables are presented as frequencies and percentages, and differences between groups were analyzed using chi-square tests. All statistical tests were two-sided, and a *p*-value < 0.05 was considered statistically significant.

## 3 Results

### 3.1 Baseline characteristics

A total of 211 patients were included in this study, comprising 133 men (63.0%) and 78 women (37.0%), with a mean age of 59.5 ± 9.1 years (range, 27–82 years). Ninety-six patients (45.5%) had a history of smoking. The main presenting symptoms were cough (*n* = 106), chest tightness (*n* = 49), fever (*n* = 24), chest pain (*n* = 18), hemoptysis (*n* = 17), and abdominal pain (*n* = 3). Comorbid conditions included hypertension (*n* = 73), diabetes (*n* = 27), coronary heart disease (*n* = 21), cerebral infarction (*n* = 16), chronic obstructive pulmonary disease (COPD) (*n* = 4), and asthma (*n* = 4).

### 3.2 Location and cases of lymph node biopsy

A total of 569 lymph node biopsies were performed on 211 patients, with station 7 lymph nodes as the most commonly sampled site, followed by station 4R lymph nodes (Table 1).

**Table 1:** EBUS-TBNA combined with EBUS-TBCB or EBUS-TBFB lymph node biopsy locations and number of cases.

Location	4R	4 L	7	10R	10 L	11R	11 L	12R
Number of biopsies	158	19	201	6	2	113	69	1

### 3.3 Sample adequacy for molecular and histopathological analyses

A total of 211 patients underwent combined EBUS-TBNA with either EBUS-TBCB or EBUS-TBFB. All biopsy specimens were systematically evaluated for their suitability for molecular testing on the basis of tissue quantity and quality. Molecular analyses were performed according to clinical indications, specimen integrity, and patient-specific factors. For benign diseases, mycobacterial DNA testing and acid-fast staining were performed, particularly in cases with granulomatous inflammation. For malignant diseases, immunohistochemistry (IHC) was initially conducted based on the initial histopathological assessment to determine tumor type and origin. Subsequently, next-generation sequencing (NGS) and PD-L1 IHC were performed on lung cancer specimens when clinically indicated.

### 3.4 Diagnostic yield

This study included 211 patients, all of whom received definitive diagnoses. Among them, 130 cases were malignant lymph node lesions, comprising 124 cases of lung cancer (119 primary and 5 metastatic) and 6 cases of lymphoma. The remaining 81 cases were benign lymph node lesions, including 53 cases of pulmonary sarcoidosis, 22 cases of lymphadenitis, and 6 cases of lymph node tuberculosis.

A total of 204 patients were successfully diagnosed using EBUS-TBNA combined with additional sampling techniques. Among them, 106 patients underwent EBUS-TBNA plus EBUS-TBCB, 65 underwent EBUS-TBA plus EBUS-TBFB, and 3 underwent both EBUS-TBCB and EBUS-TBFB in addition to EBUS-TBNA. In addition, 30 patients underwent EBUS-guided electrocautery- or laser-assisted window biopsy.

Among the 204 diagnosed patients, 121 had lung cancer, including 55 adenocarcinomas, 35 small cell carcinomas, and 24 squamous cell carcinomas. Additionally, there were 2 cases of cervical squamous cell carcinoma metastasizing to the lung, 1 case each of renal clear cell carcinoma, rectal cancer, and cardia gastric fundus adenocarcinoma metastasis to the lung, as well as 1 case of neuroendocrine tumor and 1 case of SMARCA4-deficient undifferentiated carcinoma. Other diagnoses included 50 cases of pulmonary sarcoidosis, 22 cases of lymphadenitis, 6 cases of lymph node tuberculosis, and 5 cases of lymphoma (Table 2).

**Table 2:** Sensitivity of EBUS-TBNA combined with EBUS-TBCB or EBUS-TBFB for mediastinal lymph node lesions.

Pathological type	Number of cases	Sensitivity (x/y)
Lung cancer	121	121/124
Lymphoma	5	5/6
Pulmonary nodular disease	50	50/53
Lymphadenitis	22	22/22
Tuberculosis of the lymph nodes	6	6/6

“x/y” indicates the number of cases in which a definitive diagnosis was obtained using EBUS-TBNA combined with EBUS-TBCB or EBUS-TBFB (x), relative to the total number of patients in each pathological category (y).

Among 53 patients with pulmonary sarcoidosis, 50 were confirmed by histopathology to have non-caseating epithelioid granulomatous inflammation. Combined with clinical manifestations and imaging findings (e.g., bilateral hilar lymph node enlargement), the diagnosis was established after excluding alternative causes such as tuberculosis and fungal infection. The remaining 3 cases did not yield typical pathological specimens but were diagnosed based on clinical presentation, imaging features, and response to empirical glucocorticoid therapy during follow-up. Twenty two patients with lymphadenitis were diagnosed with benign nonspecific changes on histopathology, and the final diagnoses were confirmed through clinical manifestations, imaging, and response to antibiotic therapy. Six patients with lymph node tuberculosis were diagnosed by histopathology (necrotizing granulomatous inflammation or positive acid-fast staining) and/or microbiological testing (TB-DNA PCR, Xpert MTB/RIF, or mNGS). There were four false-negative cases that were ultimately confirmed as malignant by other methods: two squamous cell carcinomas identified by follow-up bronchoscopy biopsy, one adenocarcinoma confirmed by surgery, and one lymphoma diagnosed by ultrasound-guided cervical lymph node biopsy (Table 3).

**Table 3:** Diagnostic value of combined EBUS-TBNA and EBUS-TBCB or EBUS-TBFB for benign and malignant lymph node lesions.

EBUS-TBNA combined with EBUS-TBCB or EBUS-TBFB group	Final diagnosis		Total
	+	−	
+	126	0	126
−	4	81	85
Total	130	81	211

“+” indicates a pathological diagnosis of malignancy; “−” indicates a pathological diagnosis of benignity, with the final diagnosis serving as the gold standard.

To further evaluate the diagnostic performance of EBUS-guided sampling, we assessed the accuracy of ROSE on TBNA specimens. A total of 10 false-negative ROSE cases were identified. Among these, four cases showed negative ROSE smears and negative histological findings from specimens obtained using the combined EBUS-TBNA with EBUS-TBCB or EBUS-TBFB approach. No atypical nuclei were observed on the ROSE smears, but these cases were ultimately diagnosed as malignant based on additional diagnostic procedures. The remaining six cases yielded definitive histological diagnoses using the combined sampling approach, including two lymphomas, two small cell lung cancers, one squamous cell carcinoma, and one adenocarcinoma. For malignant cases, ROSE demonstrated a sensitivity of 92.31% (120/130), a specificity of 100% (81/81), and an overall diagnostic accuracy of 95.26% (201/211), which demonstrated excellent concordance with the final cytopathological or histopathological diagnoses (Table 4).

**Table 4:** Diagnostic value of ROSE during EBUS-TBNA for benign and malignant lymph node lesions.

ROSE group	Final diagnosis		Total
	+	−	
+	120	0	120
−	10	81	91
Total	130	81	211

A ROSE result of “+” indicated the presence of cells with nuclear atypia, suggestive of a malignant diagnosis, whereas a ROSE result of “−” indicated the absence of nuclear atypia, suggestive of a benign lesion. The final pathological diagnosis was considered the reference standard.

In this study, EBUS-TBCB and EBUS-TBFB were categorized as generalized lymph node biopsy techniques, which include EBUS-guided electrocautery- or laser-assisted window biopsy, EBUS-TBCB, EBUS-TBFB, or their combination. A total of 108 cases were performed with EBUS-TBNA combined with EBUS-TBCB, 68 with EBUS-TBNA combined with EBUS-TBFB, 32 with EBUS-guided electrocautery or laser-assisted window biopsy, and 3 with EBUS-TBNA combined with both EBUS-TBCB and EBUS-TBFB. Due to the small sample size and lack of statistical power in the electrocautery/laser-assisted window biopsy group and the combined EBUS-TBCB plus TBFB group, intergroup comparisons were not conducted. Nevertheless, the overall diagnostic yield in the electrocautery/laser-assisted window biopsy group was 93.75% (30/32), and all 3 cases in the combined EBUS-TBCB and TBFB group were successfully diagnosed. No significant difference in diagnostic yield was observed between the EBUS-TBNA plus EBUS-TBCB group and the EBUS-TBNA plus EBUS-TBFB group. However, for pulmonary sarcoidosis, the diagnostic yield of EBUS-TBNA combined with EBUS-TBCB (96.67%) was higher than that of EBUS-TBNA combined with EBUS-TBFB (87.50%) (Table 5).

**Table 5:** Comparison of sensitivity between EBUS-TBNA combined with EBUS-TBCB and EBUS-TBNA combined with EBUS-TBFB.

Diagnosis	EBUS-TBNA combined with EBUS-TBCB diagnosis (x/y)	EBUS-TBNA combined with EBUS-TBFB diagnosis (x/y)	*p*-value
Malignant	59/60	46/47	1.000
Lung adenocarcinoma	24/24	21/21	
Lung squamous cell carcinoma	15/16	5/6	
Small cell lung cancer	14/14	15/15	
metastatic cancer	3/3	2/2	
Undifferentiated carcinoma	1/1	–	
Neuroendocrine carcinoma	–	1/1	
Lymphoma	3/3	2/2	
Benign	47/48	19/21	0.218
Pulmonary Nodular Disease	29/30	14/16	
Lymphadenitis	16/16	3/3	
Tuberculosis of the lymph nodes	2/2	2/2	
Total	106/108	65/68	0.376

“x/y” indicates the number of cases in which a definitive diagnosis was obtained using EBUS-TBNA combined with EBUS-TBCB or EBUS-TBFB (x), relative to the total number of patients in each pathological category (y).

### 3.5 Adverse event

In this study, no severe complications, including massive bleeding, severe hypoxemia, respiratory distress, or mediastinal infection, were observed with EBUS-TBNA combined with EBUS-TBCB or EBUS-TBFB. Minor bleeding events were effectively controlled under bronchoscopy. Specifically, three patients experienced grade 3 bleeding at the puncture site, which was successfully managed with iced saline irrigation and topical application of diluted norepinephrine, with no recurrence. Ten patients developed mild hemoptysis, presumed to result from residual airway blood, which resolved spontaneously without systemic hemostatic therapy. Two patients reported chest pain, considered a local symptom related to the biopsy site; symptoms resolved spontaneously without medication. Three patients developed transient low-grade fever (<38.5 °C), which normalized after supportive treatment, without recurrence. Four patients experienced transient postoperative cough, likely associated with general anesthesia and bronchoscope passage through the glottis, which subsided spontaneously. One patient developed mediastinal emphysema, possibly due to tissue adhesion and tearing during cryoprobe withdrawal, leading to a transmural airway wall defect and air leakage into the mediastinal connective tissue. The patient remained stable and recovered with observation and oxygen therapy, without respiratory distress or chest tightness. Overall, EBUS-TBNA combined with EBUS-TBCB or EBUS-TBFB demonstrated a favorable safety profile, with only mild, manageable adverse events observed (Table 6).

**Table 6:** Complications of EBUS-TBNA combined with EBUS-TBCB or EBUS-TBFB.

Complications	Number of cases	Rate (%)
Bleeding (Grade 3)	3	1.42
hemoptysis	10	4.74
Chest pain	2	0.95
fever	3	1.42
Cough	4	1.90
Mediastinal emphysema	1	0.47
Pneumothorax	–	–
Mediastinal infection	–	–
Shortness of breath	–	–
Hypoxemia	–	–
Vocal cord hematoma	–	–

## 4 Discussion

Intrathoracic lymph node lesions are encountered in a wide range of benign and malignant diseases, and the acquisition of adequate, high-quality biopsy specimens is essential for accurate pathological diagnosis and subsequent clinical management (13). EBUS-TBNA, as a well-established minimally invasive technique, has been widely adopted for sampling mediastinal and hilar lymph nodes. However, this approach mainly provides cytological material, and the limited amount of tissue obtained may not preserve the architectural integrity required for diagnosis. Consequently, its diagnostic efficacy can be restricted in uncommon malignancies such as lymphoma, as well as in benign conditions such as sarcoidosis and tuberculosis, where intact tissue architecture is critical for accurate interpretation (20). To address these shortcomings, EBUS-TBCB and EBUS-TBFB have been developed. These techniques often incorporate high-frequency electrocautery or laser to create a bronchial wall window, through which biopsy forceps or cryoprobes can be introduced to obtain larger, structurally preserved tissue specimens. The structural integrity of these specimens not only facilitates immunohistochemistry and molecular pathology but also markedly enhances diagnostic accuracy. Previous meta-analyses have reported an overall diagnostic yield of 90.84% for malignant tumors and 93.84% for pulmonary sarcoidosis for the diagnosis of thoracic lymph node lesions using EBUS-TBCB and EBUS-TBFB (5). In line with these findings, our study demonstrated that EBUS-TBNA combined with EBUS-TBCB or EBUS-TBFB achieved a sensitivity of 96.92% for malignant tumors and a diagnostic yield of 94.34% for pulmonary sarcoidosis, which is comparable to the efficacy reported in earlier studies. Moreover, EBUS-TBNA combined with EBUS-TBCB or EBUS-TBFB was shown to be safe, with a low complication rate and no serious adverse events observed in our cohort. Collectively, these results highlight the value of combining EBUS-TBNA with EBUS-TBCB or EBUS-TBFB as a reliable and safe diagnostic strategy for intrathoracic lymph node lesions.

EBUS-TBNA has become an essential tool for both the diagnosis and staging of lymph node metastases in non-small cell lung cancer (NSCLC). Previous studies have reported a diagnostic sensitivity of up to 93% for lymph node metastasis in primary lung cancer, and accordingly, both the American College of Chest Physicians (ACCP) and the European Respiratory Society (ERS) recommend EBUS-TBNA as the preferred technique for nodal staging in lung cancer (21–23). Compared with previous studies on the diagnostic yield of EBUS-TBNA for malignancies, our findings suggest that EBUS-TBNA combined with EBUS-TBCB or EBUS-TBFB significantly improves the overall diagnostic yield. This advantage is largely attributable to the ability of these techniques to obtain larger, structurally intact tissue specimens. Technically, the procedure relies on the puncture channel created by EBUS-TBNA, through which biopsy forceps or cryoprobes can be advanced into the lymph nodes to perform clamp or cryobiopsy, thereby obtaining larger tissue fragments. When passage of the instruments through the initial puncture channel is challenging, high-frequency electrocautery or laser can be employed to enlarge the airway wall opening (“tunnel expansion”), facilitating smooth acquisition of adequate tissue for histological assessment (24). In contrast, EBUS-TBNA depends primarily on fine needle aspiration, yielding small tissue fragments or cytological smears. Studies have indicated that only about 70% of EBUS-TBNA procedures provide core tissues suitable for histological evaluation, while the majority yield cytological material alone (25). This limitation may restrict its value in pathological diagnoses that require preservation of tissue architecture. Notably, in our cohort of 124 patients with a confirmed diagnosis of lung cancer, 121 cases were identified using the combined approach, corresponding to a sensitivity of 97.58%. These results further underscore the role of EBUS-TBCB and EBUS-TBFB as effective complementary techniques to EBUS-TBNA, contributing to improved diagnostic accuracy for lung cancer.

For benign intrathoracic lymph node lesions, the diagnostic performance of EBUS-TBNA has shown considerable variability, largely attributable to the limited tissue volume obtained and the difficulty in recognizing specific pathological features of certain benign diseases. For instance, in pulmonary sarcoidosis, the diagnostic yield of EBUS-TBNA reported in the literature ranges from 54 to 93% (26). In a randomized, multicenter trial comparing endosonography to conventional bronchoscopy for sarcoidosis diagnosis, von Bartheld et al. found that EBUS-TBNA had an 80% diagnostic yield for granulomatous inflammation, indicating good diagnostic accuracy for sarcoidosis (27). As clinical demand for accurate sarcoidosis diagnosis increases, the need for better diagnostic specimens is growing. Obtaining sufficient, structurally intact samples is essential to improve diagnostic performance. By contrast, a recent meta-analysis demonstrated that EBUS-TBCB or EBUS-TBFB could achieve a diagnostic yield as high as 93.84% (5). In our study, combining EBUS-TBNA with EBUS-TBCB or EBUS-TBFB achieved a sensitivity of 94.34% (50/53) for pulmonary sarcoidosis, which was substantially higher than the lower limit reported for EBUS-TBNA alone. This improvement is likely related to the ability of the combined approach to obtain larger and structurally intact specimens, thereby facilitating recognition of the characteristic non-caseating necrotizing epithelioid granulomatous inflammation observed in sarcoidosis. Similarly, the reported diagnostic yield of EBUS-TBNA for mediastinal lymph node tuberculosis has varied widely, ranging from 33 to 83% across studies (28). The clinical diagnosis of mediastinal lymph node tuberculosis can be challenging due to its nonspecific clinical manifestations and imaging features, yet establishing a definitive diagnosis is essential for distinguishing it from malignancy and sarcoidosis (29). By enabling the acquisition of adequate tissue specimens, the combined use of EBUS-TBNA with EBUS-TBCB or EBUS-TBFB allows for histological detection of necrotizing granulomas, thereby facilitating pathological confirmation. Moreover, when combined with molecular techniques such as Xpert MTB/RIF and TB-DNA PCR in bronchoalveolar lavage fluid (BALF), the diagnostic efficiency for lymph node tuberculosis can be further enhanced (30). In our cohort, all six patients with lymph node tuberculosis were correctly identified using the combined technique, yielding a sensitivity of 100%. However, given the limited sample size, this promising result should be interpreted with caution, and further studies with larger cohorts are warranted to validate these findings.

The diagnostic performance of EBUS-TBNA in primary or recurrent lymphoma remains limited, with an overall sensitivity of approximately 66%, and in some reports as low as 38% depending on the extent of ancillary analysis of the obtained samples (26). Nevertheless, according to the 2008 WHO Consensus on Lymphoma Diagnosis, the diagnostic yield of EBUS-TBNA is enhanced when combined with ROSE, IHC, and flow cytometry, with reported overall sensitivity reaching 77% for both primary and recurrent lymphoma (31, 32). Importantly, the diagnostic accuracy of EBUS-TBNA varies by histological subtype: it demonstrates the highest sensitivity for low-grade non-Hodgkin lymphoma (NHL), reaching up to 92%, whereas its sensitivity for Hodgkin lymphoma (HL) is substantially lower, likely due to the scarcity of Reed–Sternberg cells, which are pathognomonic for HL (32). By contrast, meta-analyses have demonstrated that EBUS-TBCB and EBUS-TBFB achieve a diagnostic yield of 93.10% for lymphoma, with high sensitivity for both newly suspected and recurrent cases (5). In our study, six patients with lymphoma were identified, of whom five were correctly diagnosed using the combined technique, resulting in a diagnostic yield of 83.33% (5/6). Although slightly lower than previously reported values, this discrepancy may be attributable to limitations inherent to the procedure, as the biopsy instrument is introduced through the puncture tract created by EBUS-TBNA, potentially restricting the range of tissue sampling and thereby reducing diagnostic adequacy. Moreover, the relatively small sample size in our cohort precluded a meaningful statistical analysis of diagnostic efficacy for lymphoma. Given that lymphoma represents only a small proportion of intrathoracic lymph node lesions, further studies with larger patient cohorts are needed to clarify and validate the clinical utility of EBUS-TBNA combined with EBUS-TBCB or EBUS-TBFB for lymphoma diagnosis.

Our study further demonstrates that the combined use of EBUS-TBNA and EBUS-TBCB or EBUS-TBFB allows the acquisition of larger, structurally preserved histological specimens, thereby substantially improving the feasibility of subsequent immunohistochemical and molecular analyses. EBUS-TBNA, as a well-established ultrasound-guided aspiration technique, facilitates repeated needle passes into the target lesion to obtain cellular and small tissue samples without the need for adjunctive electrocautery or laser assistance. In contrast, EBUS-TBCB/TBFB may require high-frequency electrocautery or laser incision of the airway wall adjacent to mediastinal lymph nodes, thereby creating a tract through which biopsy forceps or cryoprobes can be advanced to obtain larger and deeper tissue samples. Nevertheless, several limitations of EBUS-TBCB and EBUS-TBFB should be acknowledged. The limited reach of biopsy forceps and the fixed puncture angle may restrict access to certain lesion sites, and sampling from necrotic, fibrotic, or reactive hyperplastic regions can result in false-negative outcomes. Additionally, lymph nodes with thickened or fibrotic capsules pose further challenges, requiring greater technical expertise to obtain adequate tissue. These factors underscore the importance of operator experience and technique optimization to maximize diagnostic performance in clinical practice.

This study has several limitations. First, specimens obtained from both techniques were processed as combined samples rather than analyzed separately, preventing direct assessment of the individual diagnostic performance of EBUS-TBNA and EBUS-TBCB or EBUS-TBFB. As a result, the baseline diagnostic yield of EBUS-TBNA alone was derived from previously published meta-analyses. Further controlled studies are needed to directly compare the diagnostic performance of these techniques. Second, the overall sample size was relatively limited, particularly with regard to the small number of lymphoma and lymph node tuberculosis cases, which may have affected the statistical robustness of diagnostic indicators such as sensitivity and specificity. Therefore, the accuracy and safety of this combined approach require further validation through larger, multicenter prospective studies.

In summary, our findings suggest that the combination of EBUS-TBNA and EBUS-TBCB or EBUS-TBFB represents a safe and effective diagnostic approach for intrathoracic lymph node lesions. The adjunctive window-opening technique under endobronchial ultrasound guidance allows for the acquisition of sufficient and structurally intact tissue specimens, thereby significantly improving the diagnostic yield for mediastinal and hilar lymphadenopathy without increasing the risk of serious complications. Looking forward, with the continuous advancement of bronchoscopic interventional techniques, the access established by this combined approach may not only enhance diagnostic accuracy but also provide a potential pathway for precise interventional therapies in lung cancer management.

## 5 Conclusion

The combination of EBUS-TBNA and EBUS-TBCB or EBUS-TBFB demonstrated high safety and diagnostic efficacy for intrathoracic lymph node evaluation, providing sufficient tissue for molecular pathological analysis.

## Data Availability

The original contributions presented in the study are included in the article/supplementary material, further inquiries can be directed to the corresponding authors.
